# Using sentinel-2 satellite images and machine learning algorithms to predict tropical pasture forage mass, crude protein, and fiber content

**DOI:** 10.1038/s41598-024-59160-x

**Published:** 2024-04-15

**Authors:** Marcia Helena Machado da Rocha Fernandes, Jalme de Souza FernandesJunior, Jordan Melissa Adams, Mingyung Lee, Ricardo Andrade Reis, Luis Orlindo Tedeschi

**Affiliations:** 1 Department of Animal Science, Sao Paulo State University (UNESP), Campus Jaboticabal, Jaboticabal, 14884-900 Brazil; 2 Sigfarm Intelligence LLC, College Station, 77840 USA; 3 Department of Animal Science, Texas A&M University, College Station, 77843 USA

**Keywords:** Grassland ecology, Environmental sciences, Computational science

## Abstract

Grasslands cover approximately 24% of the Earth’s surface and are the main feed source for cattle and other ruminants. Sustainable and efficient grazing systems require regular monitoring of the quantity and nutritive value of pastures. This study demonstrates the potential of estimating pasture leaf forage mass (FM), crude protein (CP) and fiber content of tropical pastures using Sentinel-2 satellite images and machine learning algorithms. Field datasets and satellite images were assessed from an experimental area of Marandu palisade grass (*Urochloa brizantha sny. Brachiaria brizantha*) pastures, with or without nitrogen fertilization, and managed under continuous stocking during the pasture growing season from 2016 to 2020. Models based on support vector regression (SVR) and random forest (RF) machine-learning algorithms were developed using meteorological data, spectral reflectance, and vegetation indices (VI) as input features. In general, SVR slightly outperformed the RF models. The best predictive models to estimate FM were those with VI combined with meteorological data. For CP and fiber content, the best predictions were achieved using a combination of spectral bands and meteorological data, resulting in R^2^ of 0.66 and 0.57, and RMSPE of 0.03 and 0.04 g/g dry matter. Our results have promising potential to improve precision feeding technologies and decision support tools for efficient grazing management.

## Introduction

The ongoing growth of the human population has increased pressure on the agricultural sector. Precision farming has emerged in this global context as a fresh approach to intensively use data to improve agricultural productivity while reducing environmental effects^1^. Within agricultural context, grassland’s global importance is supported by their extent, they cover approximately 24% of the Earth’s surface and 67% of agriculturally productive land^2^. After forests, grasslands are a significant source of carbon sinks^3^ and thus play an essential role in regulating global carbon atmospheric concentration^4,5^. In addition to regulating the global carbon cycle, grasslands are the livestock industry’s primary and cheapest feed source^3^. Moreover, adequate grazing management strategies could decrease methane (a greenhouse gas) emission intensity by 22 to 35%, effectively contributing to mitigating carbon emissions from ruminant animals^6,7^. Nonetheless, efficient grazing management and sustainable pasture-based production systems require regular monitoring of pasture forage mass (FM) and nutritional value to optimize animal performance and overall productivity in grazing systems.

The performance of grazing animals is mainly driven by available forage mass, and forage quality is related to nutritive value of ingested forage (chemical composition: crude protein, CP, and fiber contents and digestibilities) and forage intake^8^. A better understanding of the nitrogen (N) content, forage dry matter (DM) mass, and chemical composition of pastures is extremely useful to support livestock managers in adjusting the stocking rate, planning adequate pasture N fertilization and supplementation to match animal needs for more sustainable production^8^. Advancements in precision livestock farming of pasture monitoring have evolved with remote sensing^9^. The advantages of remote sensing using satellites over ground-based techniques excel in providing systematic observations at different scales, from global to local, to potentially capture the spatial and temporal variability of land surfaces and retrieve historical data^10^.

Thus, the relationship between spectral reflectance from satellite optical sensors and forage mass (FM) has been investigated using vegetation indices (VI) as a proxy in regression models (e.g., linear, power, logarithmic, multiple linear) for estimating FM in temperate^11–14^ and tropical pastures^15–17^. The exponential evolution of digital computers harnessed machine learning algorithms, which have been reported to frequently enhance predictive performance compared with simpler linear regression models^17,18^. Nonetheless, in tropical pastures, the use of satellites to estimate FM has resulted in poor predictive performance^17,19^, which has been attributed to the presence of a high fraction of senescent material in the biomass and soil background scattering effects^20,21^. Therefore, the dry FM of tropical pastures still needs to be addressed and investigated to build feasible models to implement in field conditions.

Regarding nutritional attributes, hyperspectral sensors (with narrow and near-continuous spectra) and machine learning algorithms have been used to estimate the chemical parameters of different pastures with significant accuracy^22–24^. In general, those studies with hyperspectral data have shown that the most relevant wavelengths for detecting CP and fiber were in the blue, red-edge, and short infrared regions of the spectrum^16,24–26^. Due to the cost and complexity of hyperspectral sensors, the Sentinel-2 satellite, a freely available broadband multispectral satellite designed with red-edge and short infrared bands, provides an opportunity for assessing crude protein and fiber of pastures on a large scale. The European Space Agency (ESA) launched the first Sentinel-2 satellite constellation in 2015. Compared to other open sources of multispectral satellite sensors, such as Landsat and MODIS, Sentinel-2 outperforms in its spatial and temporal resolution, as well in its spectral resolution, because of the presence of red-edge bands, which were only previously incorporated in sensors of commercial satellites such as WorldView-2 and RapidEye^27^.

Previous studies highlighted the potential of Sentinel-2 spectral bands to estimate leaf N content in rangelands from South Africa^27^, fiber concentration in the seminatural grasslands of southeast Germany^28^, and CP and fiber content of Mediterranean permanent grasslands^29^ using machine learning algorithms with moderate performance and predictivity ability. Among the various machine learning algorithms, Randon forest (RF^30^;) and support vector machine (SVM^31^;) have been widely explored in remote sensing studies. Both RF and SVM are nonparametric supervised classifiers; they do not assume a known statistical distribution of the data to be classified. This is particularly relevant due to the unknown distribution of the data acquired from satellite remote sensing^31^. The main advantages of RF, a well-known regression method, are related to its ability to process high-dimensional data and prevent overfitting^32^. In contrast, SVM’s main advantages are its robustness to small training datasets and low sensitivity to free parameter settings^31^.

Therefore, the hypothesis was that spectral data from the Sentinel-2 satellite are adequate to nondestructively estimate dry FM, CP, and fiber concentrations of tropical pastures. This study aimed to estimate the dry FM, CP, and neutral detergent fiber (NDF) content of Marandu palisade grass (*Urochloa brizantha* Hochst ex A. Rich Stapf cv. Marandu) pastures using Sentinel-2 bands combined with machine learning algorithms (RF and SVM).

## Results

In this study, a field dataset and Sentinel-2 satellite images were assessed from an experimental area of Marandu palisade grass pastures, with or without nitrogen fertilization, and managed under continuous stocking. The data were gathered from January to April 2016–2020 during the pasture growing season. Models based on support vector regression (SVR) and RF machine-learning algorithms were developed using meteorological data, spectral reflectance, and VI as input features to estimate FM, CP and NDF content of tropical pastures.

## Estimation of forage mass parameters

**Figure 1:**
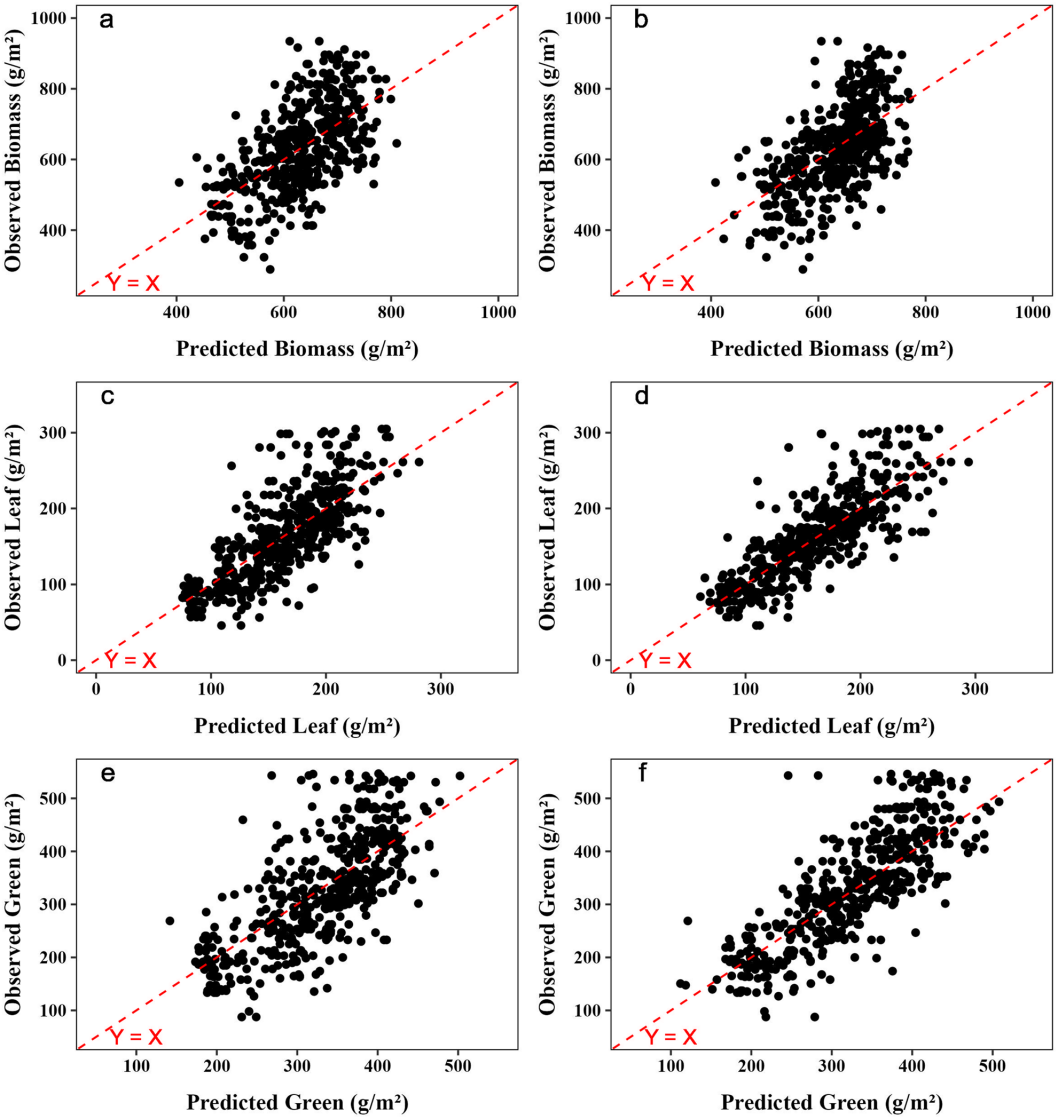
Scatterplots of the predicted versus observed values of dry forage mass using the best random forest (RF) (**a**) and support vector regression (SVR) (**b**) models, of dry leaf forage mass using the best RF (**c**) and SVR (**d**) models, and dry green (leaf + stem) forage mass using the best RF (**d**) and SVR (**f**) models.

The estimate of FM using spectral reflectance data and their VI from the Sentinel-2 satellite, with or without meteorological data, resulted in models with low to moderate precision and accuracy, with R^2^, root mean square prediction error (RMSPE), and concordance correlation coefficient (CCC) ranging from 0.20 to 0.38, 96.57 to 109.68 g/m^2^, and 0.36 to 0.54, respectively (Table 1). Otherwise, the estimate of dry forage green and leaf mass resulted in moderate to high precision and accuracy, with R^2^ and CCC ranging from 0.36 to 0.64 and 0.52 to 0.78, respectively (Table 1). No prediction bias was observed in any model (*P* > 0.10; Supplementary Table S3 online; Fig. 1).

**Table 1:** Prediction precision and accuracy of forage mass parameters (dry forage mass, dry leaf forage mass and dry green forage mass) of Marandu palisadegrass pastures using random forest and support vector regression models.

Variables	Input features^#^	Linear regression				CCC	
		R^2^		RMSPE			
	Models	RF	SVR	RF	SVR	RF	SVR
Dry forage mass (g/m^2^)	Bd	0.20 (0.05)	0.26 (0.03)	109.68 (5.37)	105.40 (6.62)	0.37 (0.03)	0.44 (0.04)
	Bd + Mt	0.35 (0.05)	0.35 (0.08)	98.77 (4.73)	100.0 (3.98)	0.52 (0.03)	0.54 (0.05)
	VI	0.20 (0.06)	0.25 (0.08)	109.49 (5.44)	106.51 (6.30)	0.36 (0.04)	0.42 (0.05)
	**VI + Mt**	**0.34** **(0.06)**	**0.37** **(0.07)**	**99.22** **(6.21)**	**97.04** **(8.81)**	**0.52** **(0.04)**	**0.52** **(0.05)**
	Bd + VI	0.23 (0.06)	0.27 (0.07)	106.95 (6.47)	105.04 (5.73)	0.38 (0.04)	0.45 (0.04)
	Bd + VI + Mt	0.34 (0.06)	0.38 (0.07)	99.81 (6.24)	96.57 (9.38)	0.50 (0.03)	0.52 (0.05)
Dry leaf forage mass (g/m^2^)	Bd	0.44 (0.08)	0.51 (0.07)	43.08 (2.19)	40.38 (2.31)	0.62 (0.06)	0.67 (0.04)
	Bd + Mt	0.53 (0.08)	0.61 (0.07)	39.31 (2.44)	36.01 (3.75)	0.68 (0.07)	0.76 (0.04)
	VI	0.45 (0.07)	0.63 (0.01)	42.74 (1.66)	35.19 (1.31)	0.62 (0.05)	0.77 (0.01)
	**VI + Mt**	**0.56** **(0.05)**	**0.62** **(0.02)**	**37.88** **(1.17)**	**35.69** **(1.88)**	**0.71** **(0.04)**	**0.78** **(0.02)**
	Bd + VI	0.46 (0.06)	0.49 (0.07)	42.11 (1.45)	41.33 (1.11)	0.64 (0.04)	0.67 (0.03)
	Bd + VI + Mt	0.56 (0.04)	0.62 (0.03)	38.26 (1.48)	35.51 (1.91)	0.70 (0.03)	0.76 (0.02)
Dry green forage mass (g/m^2^)	Bd	0.36 (0.08)	0.51 (0.06)	83.67 (5.46)	73.97 (3.85)	0.52 (0.06)	0.66 (0.04)
	Bd + Mt	0.49 (0.06)	0.58 (0.06)	74.41 (4.34)	67.67 (6.11)	0.65 (0.05)	0.73 (0.04)
	VI	0.40 (0.08)	0.50 (0.06)	80.52 (4.35)	74.82 (3.60)	0.57 (0.06)	0.64 (0.05)
	**VI + Mt**	**0.52** **(0.05)**	**0.64** **(0.03)**	**72.52** **(4.01)**	**63.48** **(3.25)**	**0.67** **(0.04)**	**0.76** **(0.02)**
	Bd + VI	0.43 (0.08)	0.52 (0.08)	79.13 (6.38)	73.41 (4.69)	0.58 (0.06)	0.66 (0.05)
	Bd + VI + Mt	0.52 (0.07)	0.63 (0.04)	72.55 (4.23)	63.54 (4.07)	0.66 (0.05)	0.76 (0.03)

The number in parenthesis represents a standard error among fivefold cross-validation. Bold represents the best models.

*RMSPE* root mean square prediction error, *CCC* concordance correlation coefficient, *RF* random forest, *SVR* support vector regression.

^#^Bd: only data from spectral bands; see Table 5 for more information; Mt: meteorological data; maximum temperature (T_max_), minimum temperature (T_min_), average temperature (T_avg_), relative humidity (RH), number of rainy days within a month (ND), rainfall; VI: only data from vegetation indices; see Table 6 for more information.

**Figure 2:**
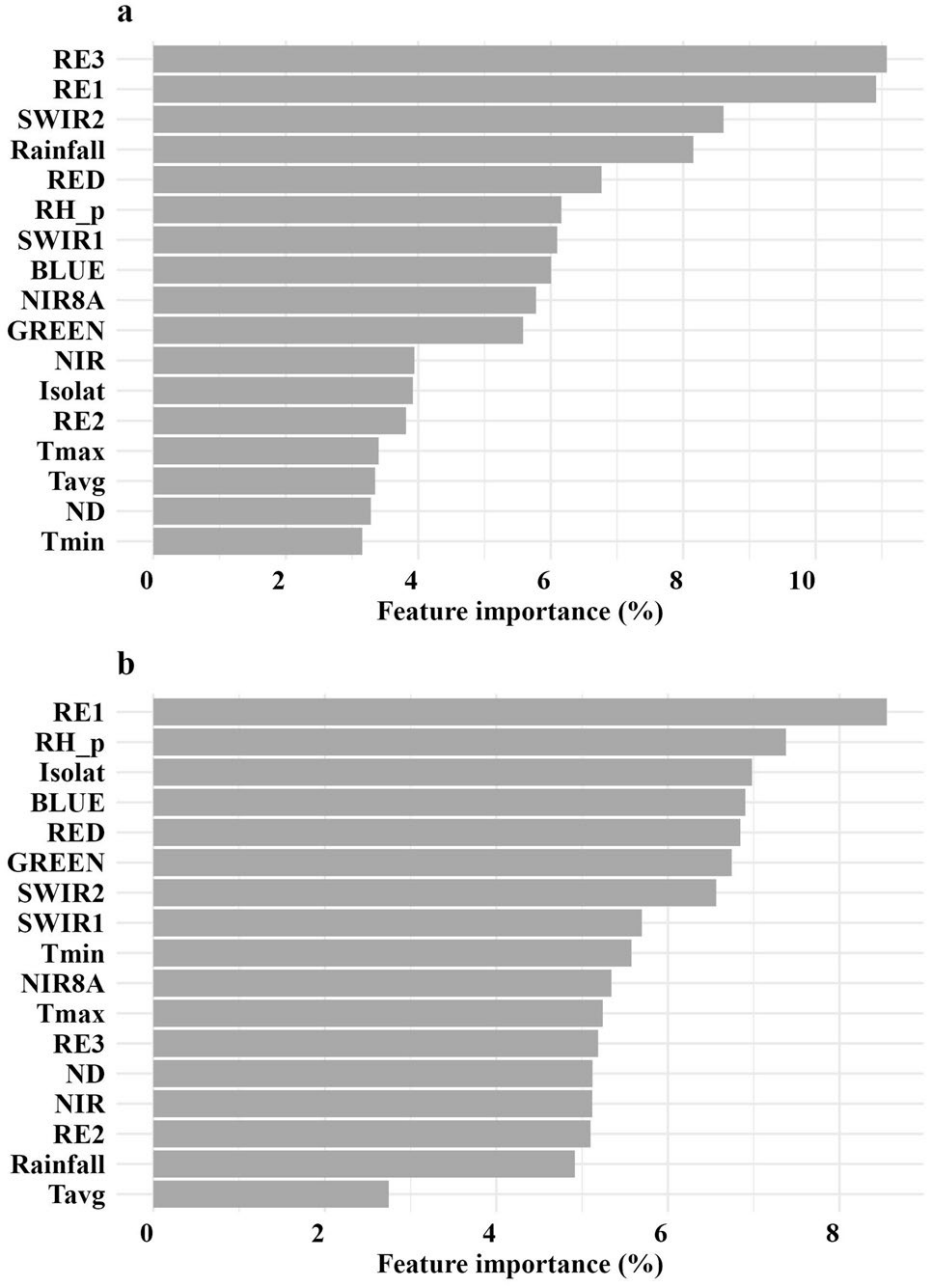
Feature importance of the best models of (**a**) dry forage mass, (**b**) dry leaf forage mass and (**c**) dry green (leaf + stem) forage mass of Marandu palisadegrass pastures. T_max_, maximum temperature; T_min_, minimum temperature; T_avg_, average temperature, RH_p, relative humidity; ND number of rainy days within a month; Isolat, insolation; CCCI, canopy chlorophyll absorption ratio index; CIgreen, chlorophyll index green; CIredge, chlorophyll index red edge; EVI, enhanced vegetation index; GDVI, normalized green difference vegetation index; GLI, green leaf index, LChloI, leaf chlorophyll index; NBR, normalized burn rate; NDVI, normalized difference vegetation index; NDVI8A, NDVI 8A; OSAVI, optimized soil adjusted vegetation index; SR, simple ratio; SRredge, simple ratio red edge.

In general, the best predictive models to estimate FM and dry leaf and green forage mass were those with VI combined with meteorological data as input features. The SVR slightly outperformed the RF models, resulting in R^2^ values of 0.37, 0.62 and 0.64 (Table 1). The main features of the best models were ranked according to their degree of importance (Fig. 2). In general, all features were of similar importance.

## Estimation of chemical composition parameters

**Figure 3:**
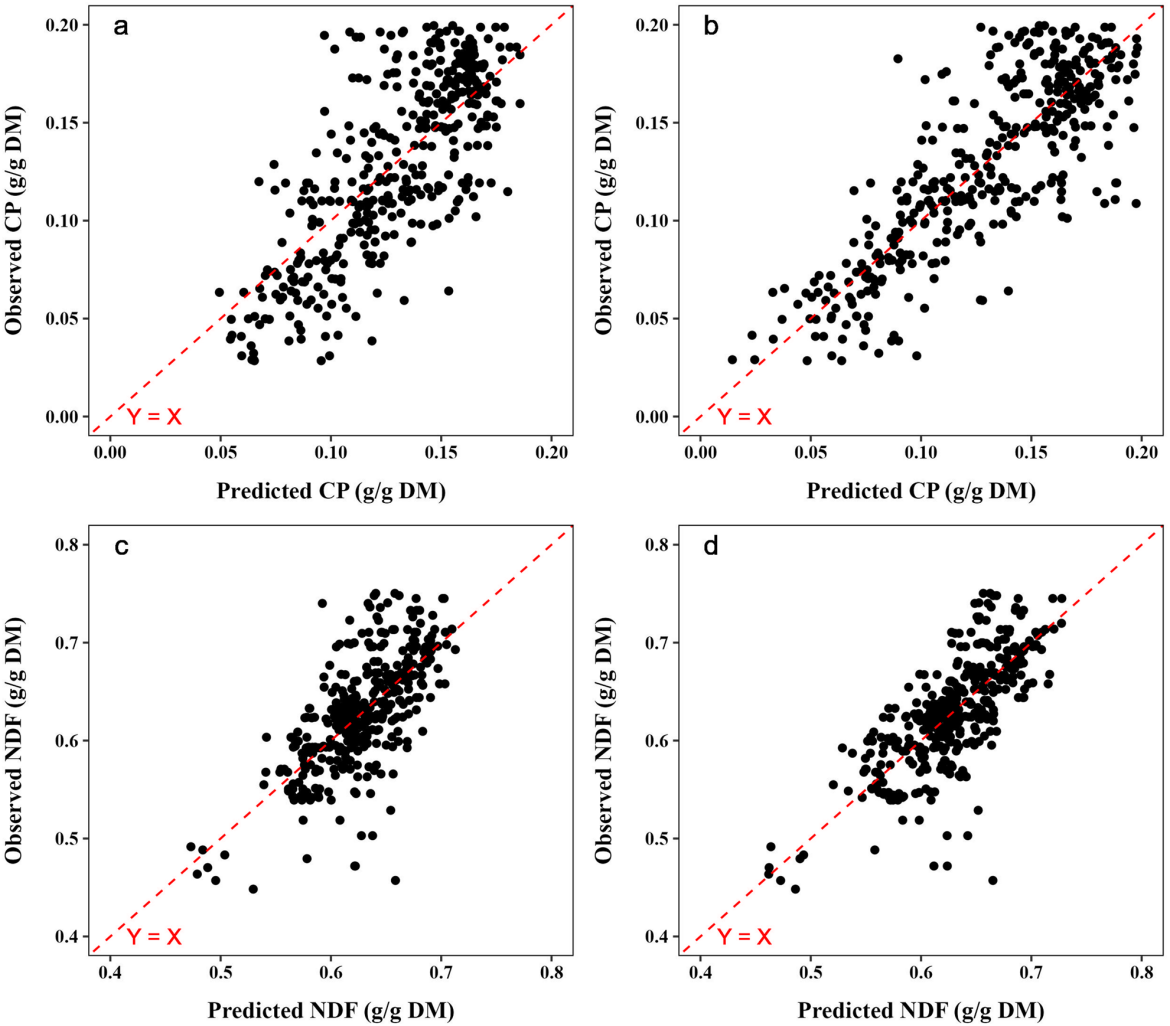
Scatterplots of the predicted versus observed values of crude protein content (CP) using the best random forest (RF) (**a**) and support vector regression (SVR) (**b**) models and neutral detergent fiber (NDF) using the best RF (**c**) and SVR (**d**) models. DM = dry matter.

For CP estimation, the highest R^2^ (0.66) and the lowest RMSPE (0.03 g/g DM) were achieved using a combination of spectral bands and meteorological data (Bd + Mt). Similarly, the Bd + Mt combination features resulted in the highest R^2^ (0.57) and the lowest RMSPE (0.04 g/g DM) for NDF estimation (Table 2). No prediction bias was observed in any model (*P* > 0.1; Supplementary Table S4 online; Fig. 3).

**Table 2:** Prediction precision and accuracy of chemical composition parameters (crude protein and neutral detergent fiber content) of Marandu palisadegrass pastures using random forest and support vector regression models.

Variables	Input features^#^	Linear regression				CCC	
		R^2^		RMSPE			
	Model	RF	SVR	RF	SVR	RF	SVR
CP (g/g DM)	Bd	0.50 (0.07)	0.64 (0.06)	0.03 (0.002)	0.03 (0.002)	0.64 (0.05)	0.79 (0.04)
	**Bd + Mt**	**0.58** **(0.09)**	**0.66** **(0.11)**	**0.03** **(0.002)**	**0.03** **(0.004)**	**0.70** **(0.06)**	**0.80** **(0.07)**
	VI	0.48 (0.04)	0.61 (0.10)	0.03 (0.001)	0.03 (0.003)	0.63 (0.03)	0.77 (0.07)
	VI + Mt	0.51 (0.12)	0.65 (0.09)	0.03 (0.003)	0.03 (0.003)	0.66 (0.07)	0.80 (0.05)
	Bd + VI	0.55 (0.05)	0.58 (0.08)	0.03 (0.001)	0.03 (0.002)	0.67 (0.03)	0.75 (0.05)
	Bd + VI + Mt	0.57 (0.10)	0.64 (0.07)	0.03 (0.002)	0.03 (0.002)	0.70 (0.06)	0.80 (0.04)
NDF (g/g DM)	Bd	0.29 (0.07)	0.36 (0.08)	0.05 (0.004)	0.05 (0.003)	0.47 (0.07)	0.54 (0.06)
	**Bd + Mt**	**0.49** **(0.07)**	**0.57** **(0.08)**	**0.04** **(0.004)**	**0.04** **(0.003)**	**0.65** **(0.06)**	**0.73** **(0.06)**
	VI	0.27 (0.07)	0.39 (0.07)	0.05 (0.003)	0.05 (0.003)	0.45 (0.07)	0.58 (0.05)
	VI + Mt	0.47 (0.09)	0.56 (0.09)	0.04 (0.004)	0.04 (0.005)	0.64 (0.07)	0.73 (0.07)
	Bd + VI	0.33 (0.04)	0.40 (0.08)	0.05 (0.002)	0.05 (0.003)	0.48 (0.04)	0.59 (0.06)
	Bd + VI + Mt	0.49 (0.07)	0.57 (0.10)	0.04 (0.003)	0.04 (0.005)	0.65 (0.05)	0.73 (0.07)

The number in parenthesis represents a standard error among fivefold cross-validation. Bold represents the best models.

*RMSPE* root mean square prediction error, *CCC* concordance correlation coefficient, *RF* random forest, *SVR* support vector regression, *CP* crude protein, *NDF* neutral detergent fiber.

^#^Bd: only data from spectral bands; see Table 3 for more information; Mt: meteorological data; maximum temperature (T_max_), minimum temperature (T_min_), average temperature (T_avg_), relative humidity (RH), number of rainy days within a month (ND), rainfall; VI: only data from vegetation indices; see Table 4 for more information.

Like forage mass, the SVR models showed slightly superior performance than the RF models in predicting CP and NDF (Table 2). The models using only VI as input variables for CP and NDF estimation showed lower precision than the other tested input feature combinations. The input of meteorological data improved the precision of CP (an increase of up to 23% in the R^2^ values) and NDF (an increase of approximately 3% in the R^2^ values) estimation when compared with using only spectral bands (Bd) or vegetation indices (VI). However, the combination of the spectral bands, vegetation indices, and meteorological data did not improve the CP and NDF estimation precision when compared to those obtained using only the Bd combined with meteorological data (Table 2).

**Figure 4:**
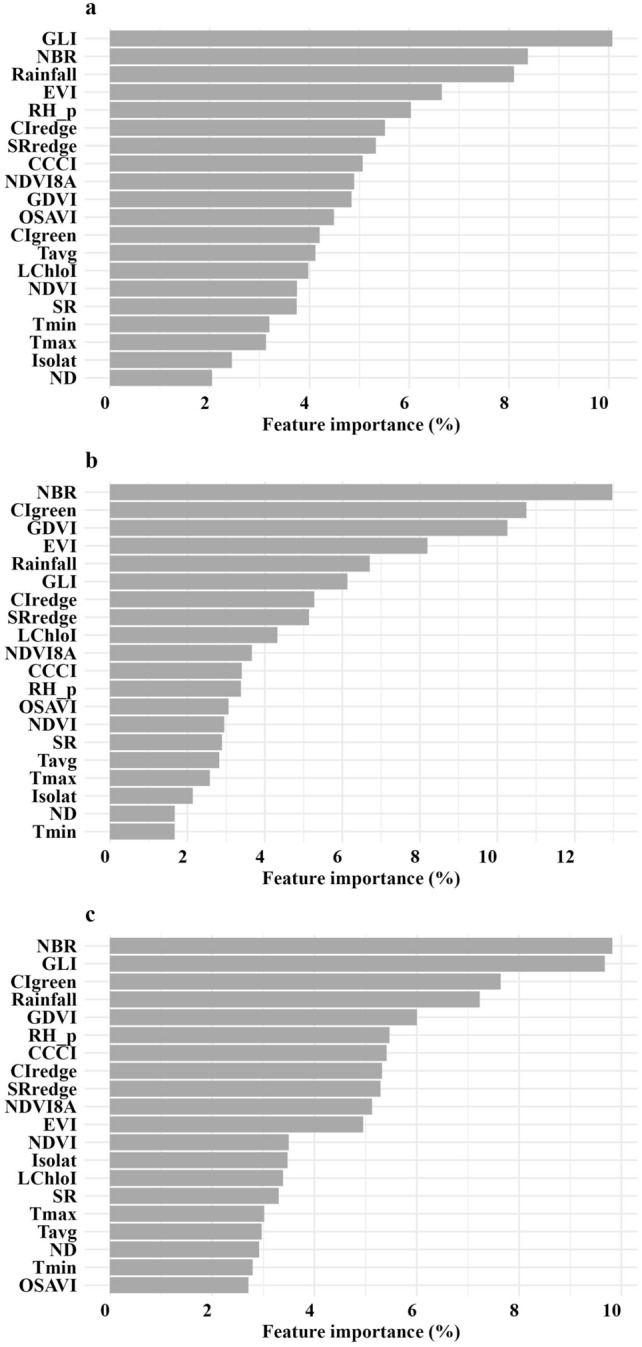
Feature importance of the best models of (**a**) crude protein and (**b**) neutral detergent fiber of Marandu palisadegrass pastures. T_max_, maximum temperature; T_min_, minimum temperature; T_avg_, average temperature, RH_p, relative humidity; ND number of rainy days within a month; Isolat, insolation; Blue (B2); Green (B3); Red (B4); RE1, Red Edge 1 (B5); RE2, Red Edge 2 (B6); RE3, Red Edge 3 (B7); NIR, Near Infrared (B8); NIR8A, Narrow NIR (B8A); SWIR1, Short Wave Infrared 1 (B11); SWIR2, Short Wave Infrared 2 (B12).

The main features of the best models were ranked according to their degree of importance (Fig. 4). In general, all features were of similar importance. Otherwise, the most influential features, above 8% of importance, were the spectral reflectances in red edges 1 and 3 for protein and red edge 1 for NDF (Fig. 4).

## Discussion

This study demonstrated the potential of estimating pasture leaf biomass and CP and NDF content of tropical pastures with moderate to high precision and accuracy using Sentinel-2 satellite images in tandem with machine learning algorithms. Such information has promising potential to improve the monitoring of tropical pasture FM and nutritive value, accounting for their spatial and temporal variability. In this sense, the Sentinel-2 satellite is a freely available broadband multispectral satellite designed with red-edge and short infrared bands. The use of red edge-based vegetation indices has been shown to improve the estimates of FM^27,33^, leaf N content^23,27,34^ and acid detergent fiber (ADF)^23^. Acquiring images from the Sentinel-2 satellite, this study also observed the importance of red edge regions in predicting the CP and NDF content of Marandu palisade grass pastures. Moreover, the inclusion of meteorological data as an input feature improved the predictive performance, elucidating the importance of rainfall and temperature in the prediction of pasture FM, as well as CP and NDF, as observed in previous studies^17,35^.

The poor performance of the models in predicting dry FM in tropical pastures has also been observed in previous studies, which reported R^2^ values less than 0.30^17,19,32^. The low predictive ability of dry FM in previous studies^17,19^ was attributed to the low variability in the dry forage mass dataset used for modeling, whose coefficient of variation was approximately 26%^19^. In the current study, the coefficient of variation for the observed dry FM dataset was approximately 19% (Table 4), which could be a plausible explanation. The other explanation for the poor prediction of dry FM in tropical pastures is related to the high presence of senescent or dead material^21^. Indeed, the proportion of dead material herein was relatively high (on average 45%, Table 2), with a coefficient of variation of approximately 30%. According to Todd et al.^11^, the loss of pigmentation from vegetation drying and senescing alters spectral reflectance characteristics, where reflectance in both visible and mid-infrared spectrum regions increases significantly. Consequently, dead and dry materials produce reflectance patterns that resemble soil. Therefore, in regions where dry or senescent biomass is a substantial canopy component, the spectral distinction between vegetation and soil background is altered, hindering FM estimation. The problem of low predictability of dry FM could be offset by using an estimate of dry leaf or green (leaf plus stem) forage mass^21^, which had relatively good model performance (R^2^ > 0.60; Table 5) in this study. Considering that the performance of grazing animals is highly correlated with the intake of leaves (the most digestible part of the plant)^4,8^, the dry leaf FM could be more representative as a proxy to include in decision-making grazing models.

Together with pasture FM, the estimates of CP and NDF content of pastures using satellite remote sensing provide an excellent opportunity for precision livestock farming to monitor forage quantity and nutritive value on large scales and with temporal variability. Attempts to estimate the N (or CP) and fiber content (NDF or ADF) of pastures have been successful using field and imaging spectroscopy^24,26^ or airborne hyperspectral data^23^, with acceptable precision (R^2^ > 0.5), because the absorption features that relate to CP and fiber (ADF^23^ and cellulose^36^) have been reported to be found at wavelengths in the red edge (705–718 nm) and shortwave infrared region of the spectrum (1400–3000 nm)^23,36^. In the literature, few studies^27–29,32^ have exploited satellite multispectral optical sensors to estimate chemical composition, which has only become possible due to the inclusion of red-edge bands in satellites such as Sentinel-2, WorldView-2, and RapidEye.

For instance, Ramoelo et al.^27^, using WorldView-2 satellite images, reported R^2^ values between 0.71 and 0.90 for models to estimate the leaf N content of grasses from rangelands of African savannas. In tropical pastures, using Sentinel-2 satellite images, Pereira et al.^32^ reported R^2^ values between 0.51 and 0.64 for models to estimate the plant N content. Likewise, Fernandez-Habaz et al.^29^ observed moderate prediction models to assess CP (R^2^ = 0.50) and NDF (R^2^ = 0.50) using the Sentinel-2 satellite in permanent grasslands from the Mediterranean region. Comparatively, the best models to estimate CP and NDF in this study showed good predictive performance, with R^2^ values of 0.66 and 0.57, RMSPE values of 0.03 and 0.04 g/g DM, and CCC values of 0.80 and 0.73, respectively. Raab et al.^28^ used Sentinel-2 and Sentinel-1 data as well as random forest regression techniques to report strong R^2^ values for ADF (0.79) and CP (0.72) forecasts. Since radar data from Sentinel-1 provide information on pasture height, which is directly proportional to the amount of cellulose and lignin present, these data could help with ADF estimation^28^. Otherwise, the authors concluded that Sentinel-2 data might be sufficient to forecast forage quality given the better homogeneity of the analyzed grasslands and the dense temporal component of their dataset, as well as the enhanced findings that could be attributed to the employment of the random forest method.

It is noteworthy that the majority of previous studies mentioned above used predictive or machine learning modeling algorithms, such as random forest^27,28,32^. Machine-learning techniques, such as RF and SVR, could be an asset in detecting the nonlinear relationship between pasture nutritive value and canopy reflectance and circumventing the overfitting and multicollinearity problem^32,37^. In this study, the SVR models slightly outperformed the RF models, presumably because SVR has shown better generalization performance when the training datasets are small^31^, as observed in this study. While RF works by ensembling multiple trees, which can lead to overfitting when data are limited, SVR’s focus on maximizing the margin can lead to more stable results on smaller datasets. Moreover, SVR models have been reported to perform better in scenarios where feature importance is unclear, such as this study (Figs. 2 and 4). While RF can compute feature importance, understanding their complex interactions can be challenging. SVR can select important features to maximize the margin, leading to a clearer understanding of feature interactions in the model^30,31^.

This study was managed with continuous stocking using put-and-take technique, whose ground data were collected monthly, and management decisions were reasonably made once or twice per month. This grazing management allowed for a gap between field collection and image availability of ± 10 days, which allowed adequate data collection free from cloud cover, which is the main limitation of satellite optical sensors. However, Bretas et al.^19^ observed that the predictive performance of the models was enhanced when the maximum interval between image acquisition and field observation was restricted to one day instead of five days. This information gap is significant in rotational stocking, where the impact of changing pasture conditions occurs in the short term during the growing season. Furthermore, previous studies suggested that the prediction ability and robustness of the models for estimating vegetation parameters may be season-specific^27,38^. Therefore, future studies aggregating data from the dry season should be performed to test and expand the applicability of the models in all seasons.

This study demonstrates the potential of estimating pasture leaf FM, CP and NDF content of tropical pastures with moderate to high precision and accuracy using Sentinel-2 satellite images in tandem with machine learning algorithms. Such information has promising potential to improve the monitoring of the quantity and nutritive value of tropical pastures, accounting for their spatial and temporal variability.

## Methods

### Study area

The study was carried out at Sao Paulo State University (UNESP), Jaboticabal, Sao Paulo State, Brazil (21°15′22″ S latitude, 48°18′58″ W longitude and 595 m elevation). The climate is humid subtropical with dry winters and warm summers (Aw), according to Köppen’s classification, and the soil is classified as a typical Hapludox with a clayey texture^39^.

**Figure 5:**
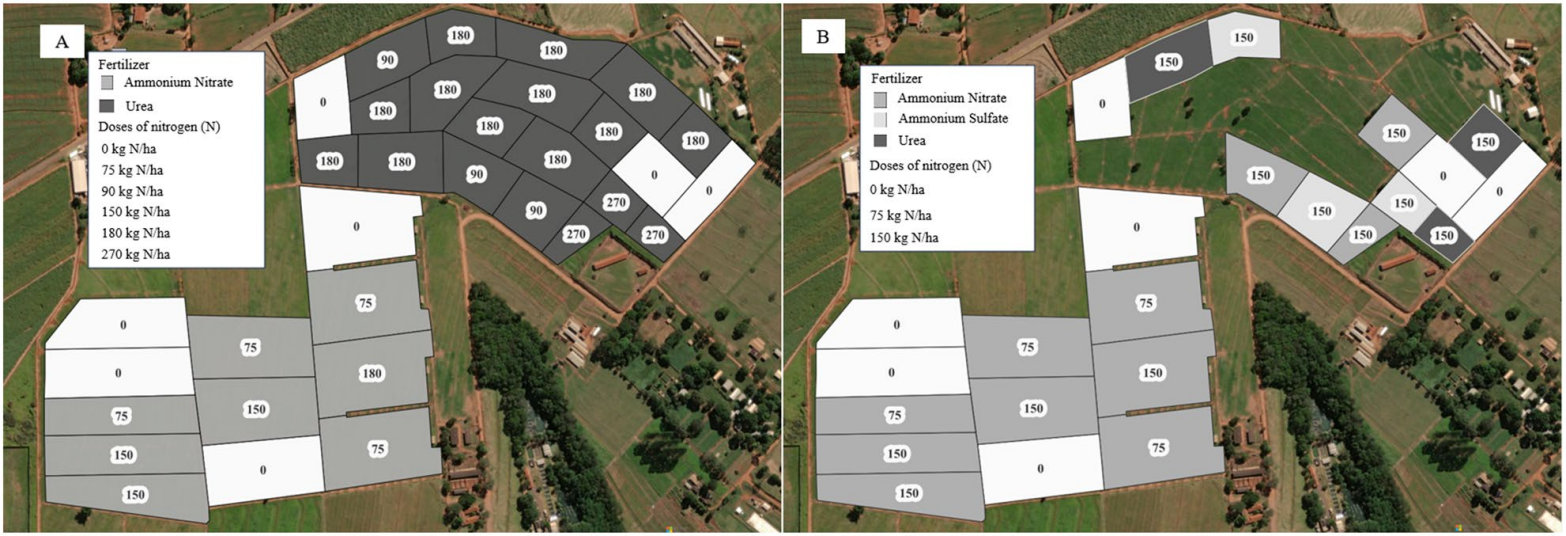
Distribution of paddocks and their nitrogen fertilization in the study area in (**A**) from 2016 to 2019 and (**B**) in 2020. 1 ha = 10.000 m^2^. The map was generated with the QGIS v.3.28.10 software (https://qgis.org/en/site/).

The site comprised 44.2 ha of pastures of Marandu palisade grass (*Urochloa brizantha* Hochst ex A. Rich Stapf cv. Marandu). From 2016 to 2019, the grazing site comprised 33 paddocks ranging from 0.5 to 2.2 ha each and was fertilized with different doses of nitrogen (N) in the form of urea (0, 90, 180, and 270 kg/ha) or ammonium nitrate (0, 75, and 150 kg/ha). In 2020, three paddocks were subdivided so that the grazing site comprised 36 paddocks ranging from 0.5 to 2.2 ha each, which received different doses of N in the form of urea (150 kg/ha), ammonium nitrate (0, 75 and 150 kg/ha) or ammonium sulfate (150 kg/ha; Fig. 5). The total amount of fertilizer was applied throughout the growing season into three applications of the same amount. We declare that no permissions or specific requirement to collect, analyze and work with *Urochloa brizantha* are required by local and national Brazilian authorities. Experimental research and field study on plant *Urochloa brizantha*, including the collection of plant material, complied with relevant institutional, national, and international guidelines and legislation.

The evaluated periods were from January to April in 2016–2020 during the pasture growing season. The mean annual rainfall was 1244 ± 186 mm, and the mean ambient temperature was 23 ± 0.3 °C. The meteorological records (Table 3) during the evaluated periods were acquired from a local meteorological station located approximately 500 m from the study area. This study was managed with continuous stocking using put-and-take technique^40^ to maintain a canopy height of 25 cm during the rainy season. The number of animals in each paddock was adjusted weekly, considering the maximum amplitude of 8% variation in canopy height (23–27 cm). The stocking rate in the paddocks varied from 1.9 to 6.5 animal units per ha (1 animal unit AU = 450 kg body weight). The animal procedures of this study were reviewed and approved by the São Paulo State University Animal Care and Use Committee guidelines and the National Council of Animal Experimentation Control (protocol approval numbers 12703/15, 7979/18, 11343/19). The procedures in this study are in accordance with ARRIVE guidelines.

**Table 3:** Meteorological data during periods of field data collection.

Month	T_max_. (°C)	T_min_. (°C)	T_avg_. (°C)	RH (%)	Rainfall (mm)	ND	Insolation (h)
2016							
Jan	29.9	20.6	24.4	82.0	449.4	18	163.5
Feb	31.9	21.1	25.2	78.8	201.0	14	180.3
Mar	31.4	20.2	24.6	79.3	132.9	17	209.0
Apr	31.5	18.0	24.0	65.6	9.1	2	267.6
2017							
Jan	30.2	20.6	24.1	80.2	217.7	19	144.7
Feb	32.0	20.0	25.0	72.5	118.3	8	236.8
Mar	31.9	19.3	24.4	71.7	126.3	11	247.3
Apr	30.0	17.9	22.8	74.7	135.7	9	217.1
2018							
Jan	30.9	20.3	24.2	78.7	270.9	21	168.2
Feb	30.5	19.7	24.0	77.4	86.8	12	186.4
Mar	32.2	20.6	25.5	73.9	55.7	10	246.8
Apr	30.8	17.3	23.2	65.9	11.0	3	238.2
2019							
Jan	32.7	20.9	26.1	69.2	148.1	11	260.6
Feb	30.9	20.4	24.4	77.5	282.6	17	160.7
Mar	31.0	20.1	24.5	76.6	115.2	12	213.2
Apr	30.6	19.0	23.9	73.4	97.6	6	228.2
2020							
Jan	31.6	20.5	24.8	79.9	350.4	20	208.0
Feb	29.8	20.3	24.0	82.9	181.1	18	123.6
Mar	31.4	18.5	23.9	70.6	101.9	6	259.1
Apr	30.1	16.1	22.5	67.2	32.6	1	257.0

Data obtained from the meteorological station of the Faculty of Agricultural and Veterinary Sciences, UNESP, Jaboticabal campus.

*T*_*max*_. maximum temperature, *T*_*min*_. minimum temperature, *T*_*avg*_. average temperature, *RH* relative humidity, *ND* number of rainy days within the month.

Detailed procedures of the grazing management of the study area for each year, as well as the field data collection, are comprehensively described in Delevatti et al.^41^, Ongaratto et al.^42^, Leite et al.^43^ and Fonseca et al.^44^.

### Field data collection

To quantify the dry FM, four samples per paddock were collected by clipping all plants at the soil level within the perimeter of a circular area of 0.25 m^2^. Samples were then separated into green leaves, dead material, and stem + sheath and dried at 55 °C to a constant weight to estimate total forage DM per hectare. Field sampling was performed periodically at intervals of 28 days. Pasture chemical composition was assessed by analyzing the hand-plucked pasture samples^45^ for N (^46^; method 984.13) and NDF^47^ content. The CP content was estimated by multiplying the N content by 6.25. Descriptive statistics of the field data collected to develop the models are depicted in Table 4.

**Table 4:** Descriptive statistics of the forage mass and chemical composition parameters of Marandu palisade grass pastures.

Variables	Count	Mean	S.D	Min	Max
Forage mass parameters					
Forage DM (g/g DM)	508	0.32	0.0897	0.12	0.62
Dry forage mass (g/m^2^)	508	631	122	289	1023
Dry leaf FM (g/m^2^)	508	162	57	46	305
Dry green FM (g/m^2^)	508	331	104	87	547
Leaf proportion (g/g DM)	508	0.26	0.078	0.12	0.45
Stem proportion (g/g DM)	508	0.26	0.073	0.12	0.47
Dead material proportion (g/g DM)	508	0.45	0.12	0.18	0.75
Chemical composition parameters					
CP (g/g DM)	399	0.129	0.0458	0.0285	0.199
NDF (g/g DM)	399	0.628	0.0576	0.448	0.750

*S.D*. standard deviation, *Min* minimum value, *Max* maximum value, *DM* dry matter, *FM*, forage mass, *CP* crude protein, *NDF* neutral detergent fiber, Green, Leaf + stem.

### Remote sensing data collection and preprocessing

All paddocks of the study area were geo-referenced to the WGS84 UTM zone 22 N map projection using an open-source image processing package (QGIS, http://www.qgis.org), and the vector layer (shapefile) was uploaded to the Google Earth Engine platform (GEE;^48^).

Using the GEE cloud platform, the spectral reflectance was obtained from the Sentinel-2 multispectral instrument product. Images were corrected to be cloud- and shadow-free over the study area. The maximum difference between field and image collection was set to 10 days. The average spectral reflectance of each Sentinel-2 band within each paddock was extracted for each image used. The average spectral reflectance of each paddock was then correlated with the data obtained in the field on dates matching the image acquisition date for attribute predictions. The bands within the Sentinel-2 satellite used in this study are depicted in Table 5.

**Table 5:** Sentinel-2 bands used in this study.

Band	Band name	Central wavelength (nm)	Spatial resolution (m)
B2	Blue	490	10
B3	Green	560	10
B4	Red	665	10
B5	Red Edge 1 (RE1)	705	20
B6	Red Edge 2 (RE2)	740	20
B7	Red Edge 3 (RE3)	783	20
B8	Near Infrared (NIR)	842	10
B8A	Narrow NIR (NIR8A)	865	20
B11	Short Wave Infrared (SWIR1)	1610	20
B12	Short Wave Infrared (SWIR2)	2190	20

Reflectance values of spectral bands were then used to calculate the vegetation indices (VI), which can reflect vegetation growth, physiological characteristics, and reduction in soil background effects (Table 6).

**Table 6:** Vegetation indices used in the estimation models.

Vegetation index^#^	Acronym	Formula
Canopy Chlorophyll Absorption Ratio Index	CCCI	$\frac{((NIR8A-RE1)/(NIR8A+RE1))}{((NIR-RED)/(NIR+RED))}$
Chlorophyll Index Green	CIgreen	$(NIR/GREEN)-1$
Chlorophyll Index Red Edge	CIredge	$(NIR8A/RE1)-1$
Enhanced Vegetation Index	EVI	$2.5 \times \frac{(NIR-RED)}{\left(NIR+6\times RED+7.5\times BLUE\right)+1}$
Normalized Green Difference Vegetation Index	GDVI	$\frac{(NIR-GREEN)}{(NIR+GREEN)}$
Green Leaf Index	GLI	$\frac{(2\times GREEN-RED-BLUE )}{(2\times GREEN+RED+BLUE )}$
Leaf Chlorophyll Index	LChloI	$\frac{(NIR-RE1)}{(NIR+RED)}$
Normalized Burn Rate	NBR	$\frac{(NIR-SWIR2)}{(NIR+SWIR2)}$
Normalized Difference Vegetation Index	NDVI	$\frac{(NIR-RED)}{(NIR+RED)}$
NDVI 8A	NDVI8A	$\frac{(NIR8A-RED)}{(NIR8A+RED)}$
Optimized Soil Adjusted Vegetation Index	OSAVI	$(1+0.16) \times \frac{(NIR-RED)}{\left(NIR+RED+0.16\right)}$
Simple Ratio	SR	$\frac{NIR}{RED}$
Simple Ratio Red Edge	SRredge	$\frac{NIR8A}{RE1}$

^#^ www.indexdatabase.de.

### Model development

**Figure 6:**
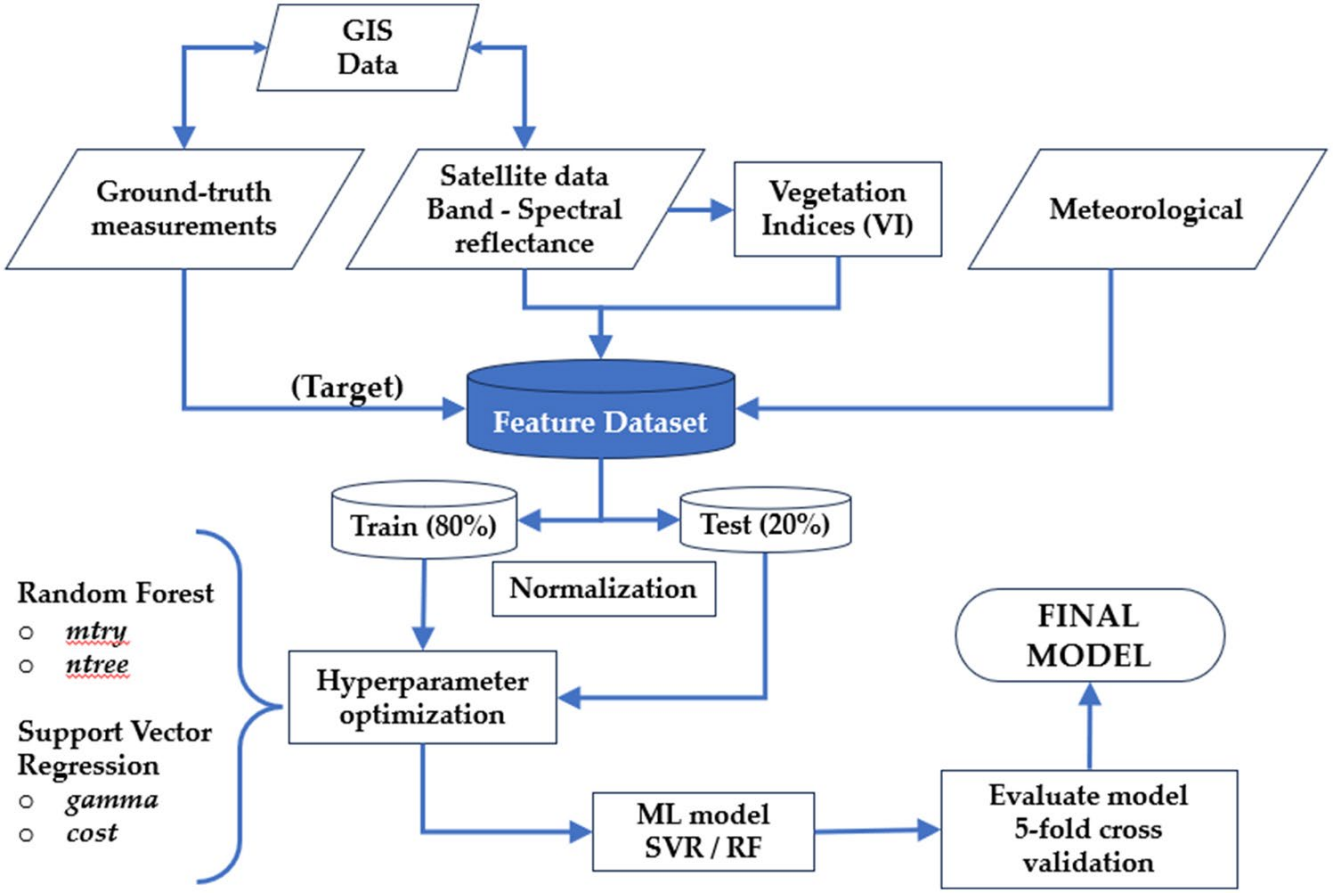
Systematic workflow of model development. SVR, support vector regression. RF, random forest.

Models based on SVR and RF machine learning algorithms were developed to estimate the dry FM, dry leaf FM, dry green (leaf + stem) FM, CP, and NDF content. The systematic workflow of this study is represented in Fig. 6.

The input features were divided into three categories: meteorological data (Mt; see Table 3), spectral reflectance of bands (Bd; see Table 5), and vegetation indices (VI; see Table 6). The potential of solely using the spectral bands or the representativeness and importance of VI, with or without meteorological data, was explored in the models as follows: Bd, only data from spectral reflectance of bands; Bd + Mt, combined spectral reflectance of bands and meteorological data; VI, only data from vegetation indices; VI + Mt, combined vegetation indices and meteorological data; Bd + VI + Mt, combined spectral reflectance of bands, vegetation indices and meteorological data.

The database was randomly split into training (80%) and testing (20%) datasets. The training dataset was used to build the models following the following steps: preprocessing data, selection of variables, and optimization of hyperparameters. After all steps, the testing dataset was applied to the final models for evaluation (Fig. 6).

*Random Forest Model:* The RF algorithm is an ensemble of decision trees based on the bagging technique. For regression problems, the RF algorithm grows many decision trees (forest), and the final prediction value corresponds to the averaged output of all individual decision trees. Each tree in the forest is independently constructed during the training process using a bootstrap sample (sample with replacement) of the training data. RF modeling was performed using R software’s ‘randomForest’ package (version 4.2.2). Developing machine learning algorithms requires a hyperparameter tuning process that maximizes the predictive accuracy of the models, whose best values depend on the research problem^49^. In this study, the optimal values of hyperparameters *mtry* (number of predictor variables randomly sampled as candidates at each split) and *ntree* (number of trees) for each model were selected according to the accuracy estimation in the training dataset using the grid-search method. In the tuning process, the candidate values ranged from 3 to 10 (square root of the total number of variables) for *mtry* and from 50 to 200 for *ntree*. The hyperparameters used in the final models are depicted in the supplementary material (Supplementary Table S1 online). Feature importance was computed from the index “Gini importance” provided by ‘randomForest’ package built-in function.

*Support Vector Regression Model:* SVR is an application of a support vector machine, which maps the input samples to a high-dimensional feature space using a nonlinear mapping function^50^, constructs a regression equation in the high-dimensional space, and then transforms the regression analysis into a quadratic programming problem, thus avoiding easily trapping local optima. In this study, the Gaussian radial basis function (RBF) kernel function, which has two hyperparameters (*gamma and cost*), was used as the core tool of SVR. The optimal values of hyperparameter *cost* and *gamma* for each model were selected using the ‘tune’ function by a grid-search method. In the tuning process, the candidate values ranged from 0.1 to 20 for *cost* and 0.001 to 1 for *gamma*. The hyperparameters in the final models are depicted in the supplementary material (Supplementary Table S2 online). SVR modeling was performed using the ‘e1071’ package of R software (version 4.2.2).

### Model evaluation

This study used a fivefold cross-validation method to evaluate the selected model because an independent evaluation dataset was unavailable. For cross-validation, the dataset was randomly divided into five subsets. For each run, four subsets were used to train the model selected in the first step, while the remaining subsets were used for prediction. The average predictive power for five iterations (fivefold cross-validation) was recorded as the final performance.

In the evaluation process, model adequacy was evaluated according to Tedeschi^51^. The precision and accuracy of all developed models were evaluated using the coefficient of determination (R^2^), root mean square prediction error (RMSPE), and concordance correlation coefficient (CCC). The CCC was classified as negligible (0.00–0.30), low (0.30–0.50), moderate (0.50–0.70), high (0.70–0.90), and very high (0.90–1.00). Residual analyses were also conducted to assess the mean and slope biases of the models. All statistical analyses for model adequacy were performed with R software (version 4.2.2). Statistical significance was declared at *P* < 0.05, and a trend was considered as 0.05 ≤ *P* < 0.1.

### Supplementary Information

Supplementary Information.

## Data Availability

The data presented in this study are fully available in this article and the Supplementary Information.
