# Supplementary material for: Using sentinel-2 satellite images and machine learning algorithms to predict tropical pasture forage mass, crude protein, and fiber content
Source: Sci Rep. 2024 Apr 15;14:8704. doi: 10.1038/s41598-024-59160-x (PMC11018762; doi:10.1038/s41598-024-59160-x)
Supplement: Supplementary file 1 — Supplementary Information. [file 41598_2024_59160_MOESM1_ESM.docx]

Using Sentinel-2 satellite images and machine learning algorithms to predict tropical pasture forage mass, crude protein, and fiber content

Marcia H M R Fernandes^*^, Jalme S. Fernandes Junior, Jordan M. Adams, Mingyung Lee, Ricardo A. Reis and Luis O. Tedeschi

*****Correspondence: Sao Paulo State University (UNESP), [marcia.fernandes@unesp.br](mailto:marcia.fernandes@unesp.br)

**Table S1.** Optimal hyperparameter *ntree* and *mtry* used in Random Forest models.

| Variables | Input features | *ntree* | *mtry* |
| --- | --- | --- | --- |
| Forage Mass parameters |  |  |  |
| Dry forage mass (g/m^2^) | Bd^1^ | 50 | 7 |
|  | Bd + Mt^2^ | 100 | 9 |
|  | VI^3^ | 150 | 5 |
|  | VI + Mt | 50 | 10 |
|  | Bd + VI | 150 | 5 |
|  | Bd + VI + Mt | 50 | 6 |
| Dry leaf forage mass (g/m^2^) | Bd | 50 | 9 |
|  | Bd + Mt | 100 | 3 |
|  | VI | 50 | 7 |
|  | VI + Mt | 150 | 7 |
|  | Bd + VI | 50 | 8 |
|  | Bd + VI + Mt | 50 | 7 |
| Dry green forage mass (g/m^2^) | Bd | 150 | 9 |
|  | Bd + Mt | 50 | 9 |
|  | VI | 150 | 8 |
|  | VI + Mt | 50 | 8 |
|  | Bd + VI | 50 | 10 |
|  | Bd + VI + Mt | 100 | 10 |
| Chemical composition parameters |  |  |  |
| CP (g/ g DM) | Bd | 200 | 10 |
|  | Bd + Mt | 100 | 5 |
|  | VI | 150 | 10 |
|  | VI + Mt | 200 | 10 |
|  | Bd + VI | 150 | 10 |
|  | Bd + VI + Mt | 50 | 10 |
| NDF (g/ g DM) | Bd | 50 | 8 |
|  | Bd + Mt | 200 | 4 |
|  | VI | 100 | 9 |
|  | VI + Mt | 50 | 6 |
|  | Bd + VI | 200 | 8 |
|  | Bd + VI + Mt | 50 | 10 |

^1^Bd: data from spectral reflectance of bands; Blue (B2), Green (B3), Red (B4), Red Edge 1 (B5), Red Edge 2 (B6), Red Edge 3 (B7), Near Infrared (B8), Narrow NIR (B8A), Short Wave Infrared 1 (B11), Short Wave Infrared 2 (B12), see Table 3 in the main text for more information.

^2^Mt: meteorological data; maximum temperature (Tmax), minimum temperature (Tmin), average temperature (Tavg), relative humidity (RH), number of rainy days within a month (ND), rainfall.

^3^VI: only data from vegetation indices; Canopy Chlorophyll Absorption Ratio Index (CCCI), Chlorophyll Index Green (CIgreen), Chlorophyll Index Red Edge (CIredge), Enhanced Vegetation Index (EVI), Normalized Green Difference Vegetation Index (GDVI), Green Leaf Index (GLI), Leaf Chlorophyll Index (LChloI), Normalized Burn Rate (NBR), Normalized Difference Vegetation Index (NDVI), NDVI 8A (NDVI8A), Optimized Soil Adjusted Vegetation Index (OSAVI), Simple ratio (SR), Simple ratio red edge (SRredge), see Table 4 in the main text for more information.

**Table S2.** Optimal hyperparameter *gamma* and *cost* used in Support Vector Regression models.

| Variables | Input features | *gamma* | *cost* |
| --- | --- | --- | --- |
| Forage Mass parameters |  |  |  |
| Dry forage mass (g/m^2^) | Bd^1^ | 1 | 5 |
|  | Bd + Mt^2^ | 1 | 5 |
|  | VI^3^ | 0.1 | 5 |
|  | VI + Mt | 0.01 | 20 |
|  | Bd + VI | 0.1 | 5 |
|  | Bd + VI + Mt | 0.01 | 15 |
| Dry leaf forage mass (g/m^2^) | Bd | 0.1 | 20 |
|  | Bd + Mt | 0.1 | 20 |
|  | VI | 0.1 | 10 |
|  | VI + Mt | 0.01 | 20 |
|  | Bd + VI | 0.1 | 10 |
|  | Bd + VI + Mt | 0.01 | 20 |
| Dry green forage mass (g/m^2^) | Bd | 0.1 | 20 |
|  | Bd + Mt | 0.1 | 20 |
|  | VI | 0.01 | 20 |
|  | VI + Mt | 0.01 | 20 |
|  | Bd + VI | 0.01 | 20 |
|  | Bd + VI + Mt | 0.01 | 20 |
| Chemical composition parameters |  |  |  |
| CP (g/ g DM) | Bd | 1 | 20 |
|  | Bd + Mt | 1 | 20 |
|  | VI | 0.1 | 10 |
|  | VI + Mt | 1 | 20 |
|  | Bd + VI | 1 | 10 |
|  | Bd + VI + Mt | 0.1 | 20 |
| NDF (g/ g DM) | Bd | 1 | 5 |
|  | Bd + Mt | 1 | 5 |
|  | VI | 0.1 | 20 |
|  | VI + Mt | 0.1 | 20 |
|  | Bd + VI | 0.1 | 15 |
|  | Bd + VI + Mt | 0.1 | 15 |

^1^Bd: data from spectral reflectance of bands; Blue (B2), Green (B3), Red (B4), Red Edge 1 (B5), Red Edge 2 (B6), Red Edge 3 (B7), Near Infrared (B8), Narrow NIR (B8A), Short Wave Infrared 1 (B11), Short Wave Infrared 2 (B12), see Table 3 in the main text for more information.

^2^Mt: meteorological data; maximum temperature (Tmax), minimum temperature (Tmin), average temperature (Tavg), relative humidity (RH), number of rainy days within a month (ND), rainfall.

^3^VI: only data from vegetation indices; Canopy Chlorophyll Absorption Ratio Index (CCCI), Chlorophyll Index Green (CIgreen), Chlorophyll Index Red Edge (CIredge), Enhanced Vegetation Index (EVI), Normalized Green Difference Vegetation Index (GDVI), Green Leaf Index (GLI), Leaf Chlorophyll Index (LChloI), Normalized Burn Rate (NBR), Normalized Difference Vegetation Index (NDVI), NDVI 8A (NDVI8A), Optimized Soil Adjusted Vegetation Index (OSAVI), Simple ratio (SR), Simple ratio red edge (SRredge), see Table 4 in the main text for more information.

**Table S3.** Prediction bias (% RMSPE) of forage mass parameters (dry forage mass, dry leaf forage mass and dry green forage mass) of Marandu palisadegrass pastures using random forest and support vector regression models.

|  |  | Bias (% RMSPE) |  |  |  |  |  |
| --- | --- | --- | --- | --- | --- | --- | --- |
| Variables^2^ | Input features | Mean bias |  | Slope bias |  | Random Bias |  |
|  | Model | RF | SVR | RF | SVR | RF | SVR |
| Forage mass parameters |  |  |  |  |  |  |  |
| Dry forage mass (g/m^2^) | Bd | 0.21 | 0.29 | 2.64 | 1.77 | 97.15 | 97.94 |
|  | Bd + Mt | 0.51 | 0.56 | 1.82 | 4.58 | 97.67 | 94.85 |
|  | VI | 0.30 | 0.44 | 2.30 | 2.51 | 97.40 | 97.05 |
|  | VI + Mt | 0.61 | 0.60 | 1.88 | 1.83 | 97.50 | 97.57 |
|  | Bd + VI | 0.27 | 0.95 | 1.69 | 2.56 | 98.04 | 96.49 |
|  | Bd + VI + Mt | 0.43 | 0.53 | 1.93 | 1.49 | 97.65 | 97.97 |
| Dry leaf forage mass (g/m^2^) | Bd | 1.06 | 1.62 | 1.65 | 1.52 | 97.30 | 96.86 |
|  | Bd + Mt | 0.60 | 0.33 | 0.58 | 1.68 | 98.81 | 97.98 |
|  | VI | 1.70 | 0.66 | 1.46 | 1.07 | 96.84 | 98.26 |
|  | VI + Mt | 0.64 | 1.48 | 1.08 | 1.48 | 98.28 | 97.03 |
|  | Bd + VI | 1.38 | 2.77 | 1.18 | 3.19 | 97.43 | 94.04 |
|  | Bd + VI + Mt | 0.85 | 0.85 | 0.87 | 0.94 | 98.28 | 98.21 |
| Dry green forage mass (g/m^2^) | Bd | 0.56 | 3.15 | 1.32 | 1.82 | 98.12 | 95.02 |
|  | Bd + Mt | 0.85 | 2.90 | 1.06 | 0.35 | 98.09 | 96.74 |
|  | VI | 0.90 | 3.69 | 0.70 | 2.35 | 98.40 | 93.96 |
|  | VI + Mt | 0.61 | 3.25 | 1.14 | 1.09 | 98.25 | 95.66 |
|  | Bd + VI | 1.00 | 3.49 | 1.10 | 2.87 | 97.90 | 93.63 |
|  | Bd + VI + Mt | 0.93 | 3.24 | 2.14 | 0.60 | 96.93 | 96.15 |

RMSPE, root mean square prediction error; RF, random forest; SVR, support vector regression.

^1^Bd: only data from spectral bands; see Table 3 in the main text for more information.

^2^Mt: meteorological data; maximum temperature (Tmax), minimum temperature (Tmin), average temperature (Tavg), relative humidity (RH), number of rainy days within a month (ND), rainfall.

^3^VI: only data from vegetation indices; see Table 4 in the main text for more information.

**Table S4.** Prediction bias (% RMSPE) of chemical composition parameters (crude protein and neutral detergent fiber content) of Marandu palisadegrass pastures using random forest and support vector regression models.

|  |  | Bias (% RMSPE) |  |  |  |  |  |
| --- | --- | --- | --- | --- | --- | --- | --- |
| Variables | Input features | Mean bias |  | Slope bias |  | Random Bias |  |
|  | Model | RF | SVR | RF | SVR | RF | SVR |
| Chemical composition parameters |  |  |  |  |  |  |  |
| CP (g/ g DM) | Bd | 1.08 | 0.55 | 1.71 | 2.09 | 97.21 | 97.35 |
|  | Bd + Mt | 1.60 | 0.53 | 2.84 | 4.40 | 95.56 | 95.07 |
|  | VI | 0.81 | 1.13 | 0.64 | 2.72 | 98.54 | 96.15 |
|  | VI + Mt | 1.19 | 0.90 | 2.36 | 3.78 | 96.46 | 95.32 |
|  | Bd + VI | 0.80 | 1.00 | 2.90 | 3.29 | 96.29 | 95.71 |
|  | Bd + VI + Mt | 1.09 | 0.39 | 2.48 | 4.63 | 96.43 | 94.98 |
| NDF (g/ g DM) | Bd | 1.45 | 2.64 | 0.45 | 1.82 | 98.10 | 95.54 |
|  | Bd + Mt | 2.29 | 1.42 | 0.10 | 0.96 | 97.61 | 97.62 |
|  | VI | 1.69 | 3.00 | 1.44 | 1.94 | 96.87 | 95.07 |
|  | VI + Mt | 1.54 | 2.06 | 0.64 | 0.81 | 97.82 | 97.14 |
|  | Bd + VI | 1.72 | 1.98 | 0.38 | 2.07 | 97.90 | 95.95 |
|  | Bd + VI + Mt | 2.14 | 1.52 | 0.23 | 1.45 | 97.63 | 97.02 |

RMSPE, root mean square prediction error; RF, random forest; SVR, support vector regression.

^1^Bd: only data from spectral bands; see Table 3 in the main text for more information.

^2^Mt: meteorological data; maximum temperature (Tmax), minimum temperature (Tmin), average temperature (Tavg), relative humidity (RH), number of rainy days within a month (ND), rainfall.

^3^VI: only data from vegetation indices; see Table 4 in the main text for more information.
